# Surgery for lung cancer: postoperative changes and complications—what the Radiologist needs to know

**DOI:** 10.1186/s13244-021-01047-w

**Published:** 2021-08-12

**Authors:** Julien Burel, Mathias El Ayoubi, Jean-Marc Baste, Matthieu Garnier, François Montagne, Jean-Nicolas Dacher, Matthieu Demeyere

**Affiliations:** 1 Service de Radiologie, Hôpital Charles-Nicolle, CHU de Rouen, 37 boulevard Gambetta, Rouen, France; 2 Service de Chirurgie Thoracique, Hôpital Charles-Nicolle, CHU de Rouen, 37 boulevard Gambetta, Rouen, France

**Keywords:** Chest computed tomography, Thoracic surgery, Lung neoplasms

## Abstract

Imaging findings after thoracic surgery can be misleading. Knowledge of the normal post-operative anatomy helps the radiologist to recognise life-threatening complications and conversely not to wrongly evoke a complication in cases of trivial post-operative abnormalities. In this educational article, we reviewed the expected patterns after thoracic surgery including sublobar resection, lobectomy, pneumonectomy and related techniques. Imaging aspects of frequent and less common complications and their typical imaging features are then presented.

## Key points

- Chest computed tomography is helpful in the post-operative assessment of lung cancer.
- Imaging findings after thoracic surgery can be misleading.
- Identifying and categorizing post-operative changes and complications can reflect positively on a patient’s prognosis.

## Background

Worldwide, lung cancer is the leading cause of cancer death. In 2018, GLOBOCAN estimated 2.09 million new cases (11.6% of total cancer cases) and 1.76 million deaths (18.4% of total cancer deaths), and these numbers are rising [1, 2].

Surgery is considered the treatment of choice for individuals with stage I and II non‐small cell lung cancer. Stage III patients are usually treated with a combination of radiotherapy and chemotherapy. Stage IV patients are treated exclusively with systemic therapy, which may include chemotherapy, targeted therapy and/or immunotherapy [3, 4].

More than 50% patients are diagnosed with stage IV disease which 5-year survival rate is around 1% [5]. In the future, lung cancer chest computed tomography (CT) screening may allow early stage detection leading to an increasing ratio of patients eligible to curative surgery [6].

As most of these patients will be followed up using chest CT to detect recurrence and complications, it appears crucial for the radiologist to be well aware of post-operative findings.

## Surgical techniques

Surgical procedures include sublobar resection, lobectomy, sleeve lobectomy and pneumonectomy.

Two types of sublobar resection are being used: non-anatomic sublobar resection, known as wedge resection, and segmentectomy, a limited resection based on the segmental vessels and airways.

Wedge resection is chosen in patients with poor pulmonary reserve and is associated with a decreased rate of complications [7].

Segmentectomy is another alternative to lobectomy, associated with larger parenchyma margins and higher yield of lymph nodes than wedge resection [8].

Lobectomy is known as the optimal technique for lung cancer. It preserves lung function compared to pneumonectomy and carries a mortality rate of 2–4% [9–11].

Pneumonectomy is indicated when cancer involves proximal bronchi or vascular structures, with a mortality rate of 6–8% [9–11].

Sleeve lobectomy is used when lung cancer invades the main bronchus: the lobe and the affected portion of the airway are resected, the proximal and distal edges of the remaining bronchus are reattached with an end-to-end anastomosis [12].

Each of these techniques can lead to complex post-operative changes on chest CT. The latter should not be misdiagnosed as complications which are also possible.

Communication between the radiologist and the surgeon about the technique used and operative findings appears essential as each technique carries specific normal and pathological aspects on chest CT.

## Role of minimally invasive resections

Although most of lung resections involve open surgery techniques (posterolateral thoracotomy, anterolateral thoracotomy, rarely median sternotomy), minimally invasive techniques (video-assisted thoracoscopic surgery, robot-assisted thoracoscopic surgery) have recently emerged as an alternative for selected patients.

Video-assisted thoracoscopic surgery is a safe and less morbid alternative to open resection, but doubt remains about its oncological equivalence. When compared to thoracotomy, it is associated with a lower incidence of post-operative complications, a shorter hospital stay, shorter duration of chest tube drainage and comparable or lower rates of post-operative mortality [13].

Robot-assisted thoracoscopic surgery has the theoretical advantages of three-dimensional visualisation and increased rotational capabilities compared to video-assisted thoracoscopic surgery. There are no differences in short-term outcomes between these two minimally invasive techniques. Improvements in immediate outcomes, such as length of stay, bleeding, duration of chest tube drainage and perioperative mortality were demonstrated when robot-assisted thoracoscopic surgery was compared to open resection. However, the increased associated costs represent a significant deterrent of this technique [13].

## Imaging follow-up

Imaging is essential in early post-operative follow-up and in the characterisation of complications. Chest radiograph (CXR) is sufficient in patients’ regular follow-up, chest CT is needed when a complication is suspected.

CT protocol to address complications of lung cancer surgery includes intravenous administration of iodinated contrast medium. An appropriate dual-phase protocol, including arterial and delayed phases, may be useful for detecting both vascular and pleural complications [14]. Split-bolus contrast injection protocol may help reduce radiation exposure in selected patients [15]. Maximum intensity projection (MIP) algorithm helps analyse vascular complications. Minimum intensity projection (MinIP) facilitates the detection of the bronchial ones. Assessment of air trapping may require additional dynamic or end-expiratory CT acquisitions.

## Review

### Normal post-operative anatomy

**Fig. 1:**
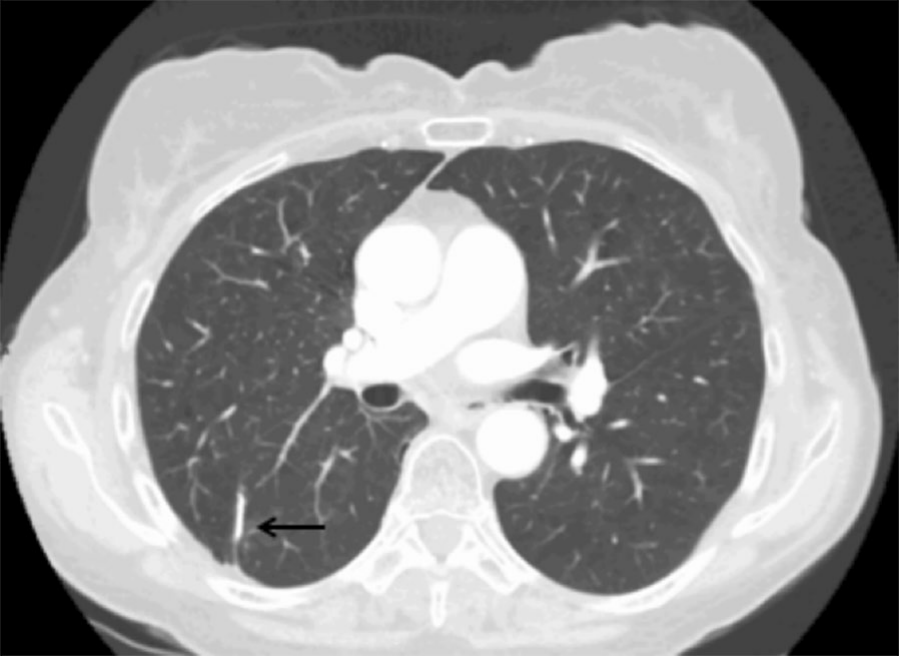
Wedge resection. Axial CT image (parenchymal window) shows a linear increased density due to a parenchymal staple line (black arrow)
B. **Lobectomy and bilobectomy**
  One or two lobes are resected, resulting in a significant loss of volume, with compensation by expansion and reorientation of the remaining lobe(s), associated with mediastinal deviation and displacement of the bronchovascular hilar structures. Post-operative monitoring consists of a daily CXR, on which the positioning of the drain(s) is evaluated, as well as the re-expansion of the remaining homolateral lobe(s) (Fig. 2) [16].

A. **Wedge resection and segmentectomy**
  Wedge resection is a non-anatomic sublobar resection, whereas segmentectomy corresponds to an anatomic sublobar resection of one to four segments. CXR and CT show a linear increased density due to a parenchymal staple line (Fig. 1). At early stage, a small area of peripheral ground-glass opacities and consolidation can be observed on CT images, reflecting contusion and haemorrhage.

**Fig. 2:**
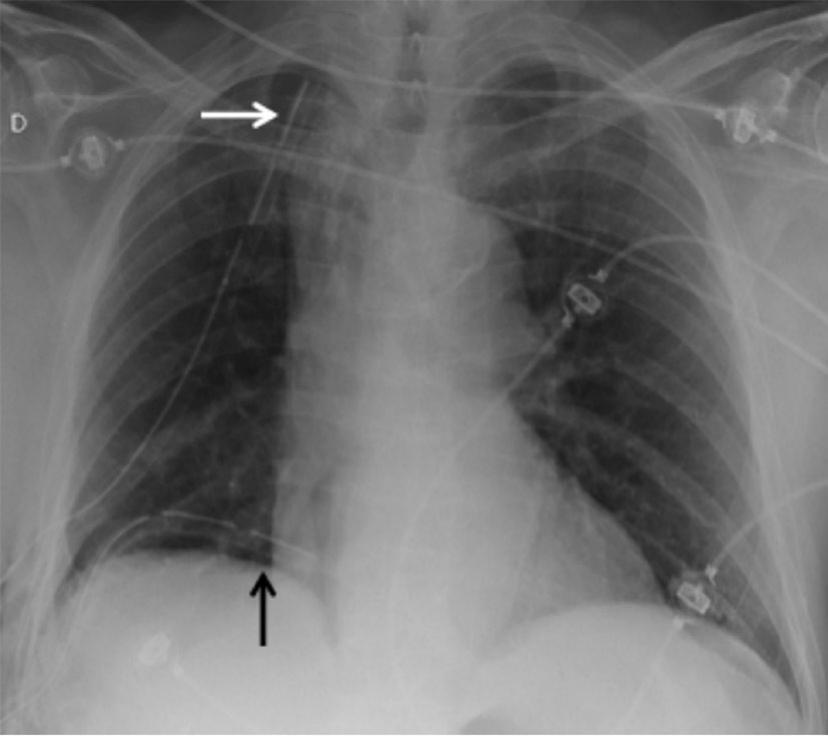
Post-operative radiograph of a right lower lobe resection. CXR allows visualisation of the drains in postero-basal (black arrow) and antero-apical (white arrow) positions
  The early post-operative aspect consists in a pneumothorax or hydropneumothorax of low abundance, parietal emphysema and retraction of the hemithorax. After a few days and weeks, a decrease in the signs of retraction and an expansion of the remaining lobe(s) appear. Later on, imaging shows retraction of the operated hemithorax, mediastinal attraction, filling of the costo-diaphragmatic recess, elevation of the diaphragmatic dome, hilar displacement and pulmonary hyperlucency (related to compensatory hyperinflation with relative vascular rarefaction); parietal sequelae and surgical material persist. Chest X-ray can demonstrate juxta-phrenic peak sign or a pseudo-lobar collapse aspect that can be misleading for atelectasis if the operating history is not known by the radiologist (Fig. 3) [17].

**Fig. 3:**
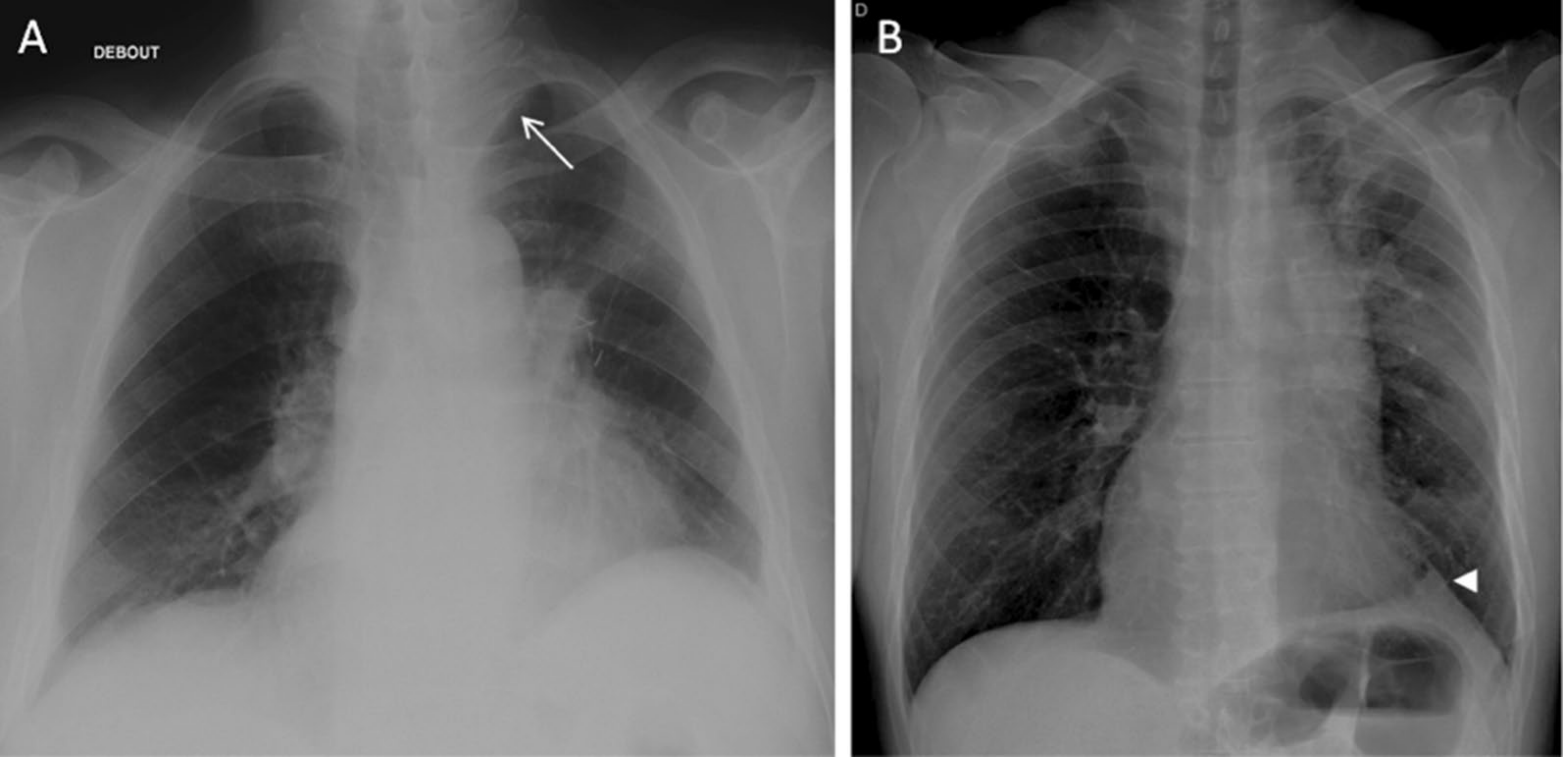
Lobar pseudo-collapse and juxta-phrenic peak sign.** A**: CXR showing a left upper lobar pseudo-collapse (white arrow).** B**: right juxta-phrenic peak sign (white arrowhead)
  Scissure changes and displacement may vary according to the site of lobectomy.
  Left upper lobectomy: imaging shows an ascension of the lower lobe with its scissural aspect facing the anterior upper mediastinum where a fluid collection can appear and persist permanently or be secondarily replaced by fibrous tissue.
  Left lower lobectomy: downward and backward movement of the lingula.
  Right upper lobectomy: upward displacement of the middle lobe and the apical segment of the lower lobe, revealing a frontally oriented cranial neoscissure in the extension of the initial oblique scissure.
  Right middle lobectomy: slight loss of lung volume, with merging of the upper and lower lobes, revealing an oblique caudal neoscissure.
  Right lower lobectomy: downward displacement of the dorsal segment of the upper lobe and rearward displacement of the middle lobe, showing an axially oriented posterior neoscissure; there may be a notable variation, with a greater displacement of the dorsal segment of the upper lobe extending to the diaphragm.
  Bilobectomy: the loss of volume is much greater, with mediastinal deviation on the operated side and diaphragmatic ascent; in case of missing information, it is possible to identify the residual lobe by looking at the vessels and the bronchial tree (Fig. 4).

**Fig. 4:**
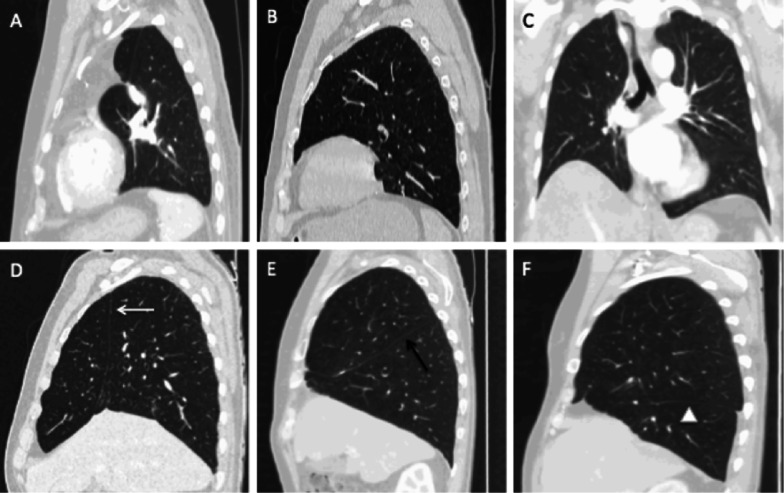
Scissure changes and normal displacement after lobectomy (parenchymal windowing). **A**: Sagittal CT image of a left upper lobectomy showing hyperinflated left lower lobe and mediastinal fat attraction without scissure. **B**: Sagittal CT image of a left lower lobectomy, note the absence of the oblique scissure. **C**: Coronal CT image after right lower and middle bilobectomy demonstrating elevated right hemidiaphragm and no visible scissure. **D**: Sagittal CT image of a right upper lobectomy with frontally oriented cranial neoscissure (white arrow). **E** Sagittal CT image after right middle lobectomy with oblique caudal neoscissure (black arrow). **F**: Sagittal CT image after right lower lobectomy with axially oriented posterior neoscissure (white arrowhead)
C. **Pneumonectomy**
  Corresponds to the resection of an entire lung with or without resection of the parietal pleura. Post-operative monitoring consists of a daily CXR that visualises the evolution of the pneumonectomy cavity. In the early post-operative period, an air-filled cavity with parietal and sometimes mediastinal emphysema is visualised in the immediate aftermath, with the trachea and mediastinum in median position. A progressive accumulation of liquid is observed with the appearance of an air–fluid level. The cavity fills at a rate of about two intercostal spaces per day. The key point to remember is that the air–fluid level reaches the upper half or two-thirds of the hemithorax in 4 to 7 days. Complete filling occurs after a few weeks or months. Absence of filling or decrease in the level should raise concern for complication. The trachea and mediastinum gradually move to the operated side, with ascent of the diaphragmatic dome and intercostal pinch on the same side. The contralateral lung undergoes compensatory expansion and becomes hypoattenuating due to vascular rarefaction (Fig. 5) [17].

**Fig. 5:**
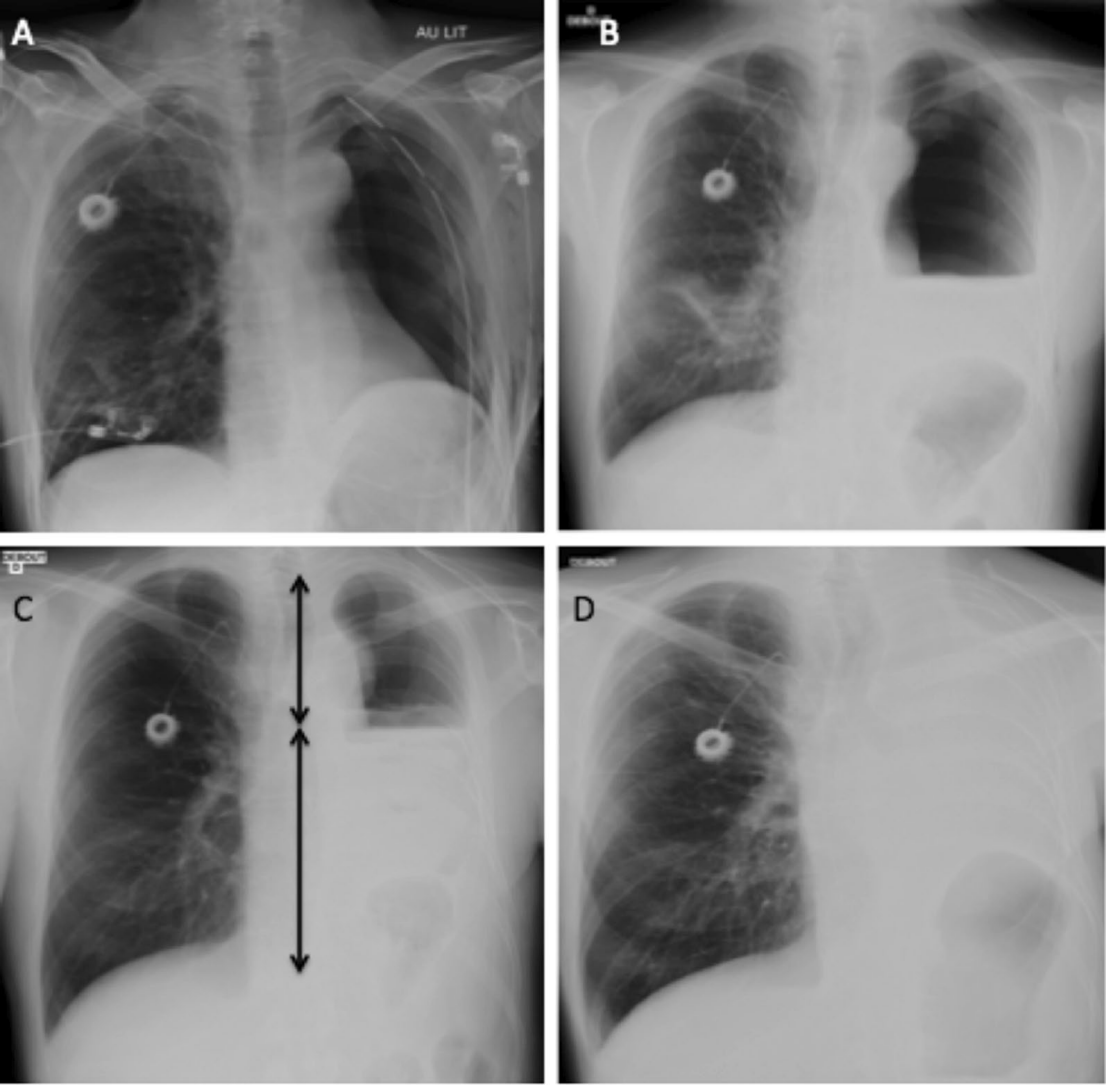
Pneumonectomy cavity evolution. **A**: First post-operative CXR in the hours following left pneumonectomy, with a chest tube in antero-apical position. **B**: Day 3 CXR with progressive filling of the pneumonectomy cavity. **C**: Day 7 CXR on which you can see that the air–fluid level reaches two-thirds of the pneumonectomy cavity. **D**: CXR after 2 months showing a complete filling of the cavity
  Later, the pulmonary arterial stump may be the site of an alluvial thrombus (Fig. 6), generally non-pathological if it is not progressive. A long bronchial stump may be the site of an accumulation of secretions of fluid like and sometimes tissular attenuation on unenhanced chest CT. In such case, contrast uptake would suggest local recurrence or superimposed infection. Mediastinal deviation on the side of the pneumonectomy is seen at variable degrees. Possible parietal sequelae that can consist of costal reshaping, herniation of the parietal fat at the site of thoracic wall resection, lymph node removal clips and possibly a high attenuation Goretex prosthesis can be present in case of diaphragmatic surgery.

**Fig. 6:**
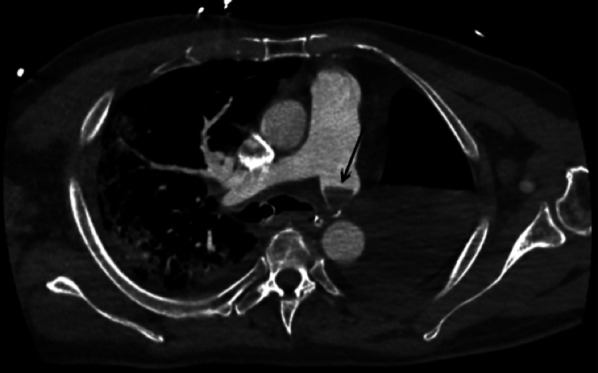
Alluvial thrombus. Axial CT image (mediastinal window) showing an alluvial thrombus in the left pulmonary arterial stump (black arrow)
  An interposition flap may cover the bronchial stump and show fatty attenuation.
  Finally, normal evolution of the post-pneumonectomy space can be towards complete resorption or the persistence of a residual pouch.
D. **Sleeve lobectomy**
  It is an alternative to pneumonectomy when the tumour invades the origin of a lobar bronchus or when N1 nodes are fixed at the origin of the lobar bronchus to be resected. This technique has the advantage of preserving a better inspiratory reserve than pneumonectomy. An enlarged lobectomy is performed to remove the invaded bronchial division with reimplantation of the remaining lobe on the main bronchus via a termino-terminal anastomosis, with or without arterial reimplantation. This surgery mostly involves the right upper lobe because of the advantage of the relative length of the intermediate bronchus allowing for reimplantation (Table 1, Fig. 7).

**Fig. 7:**
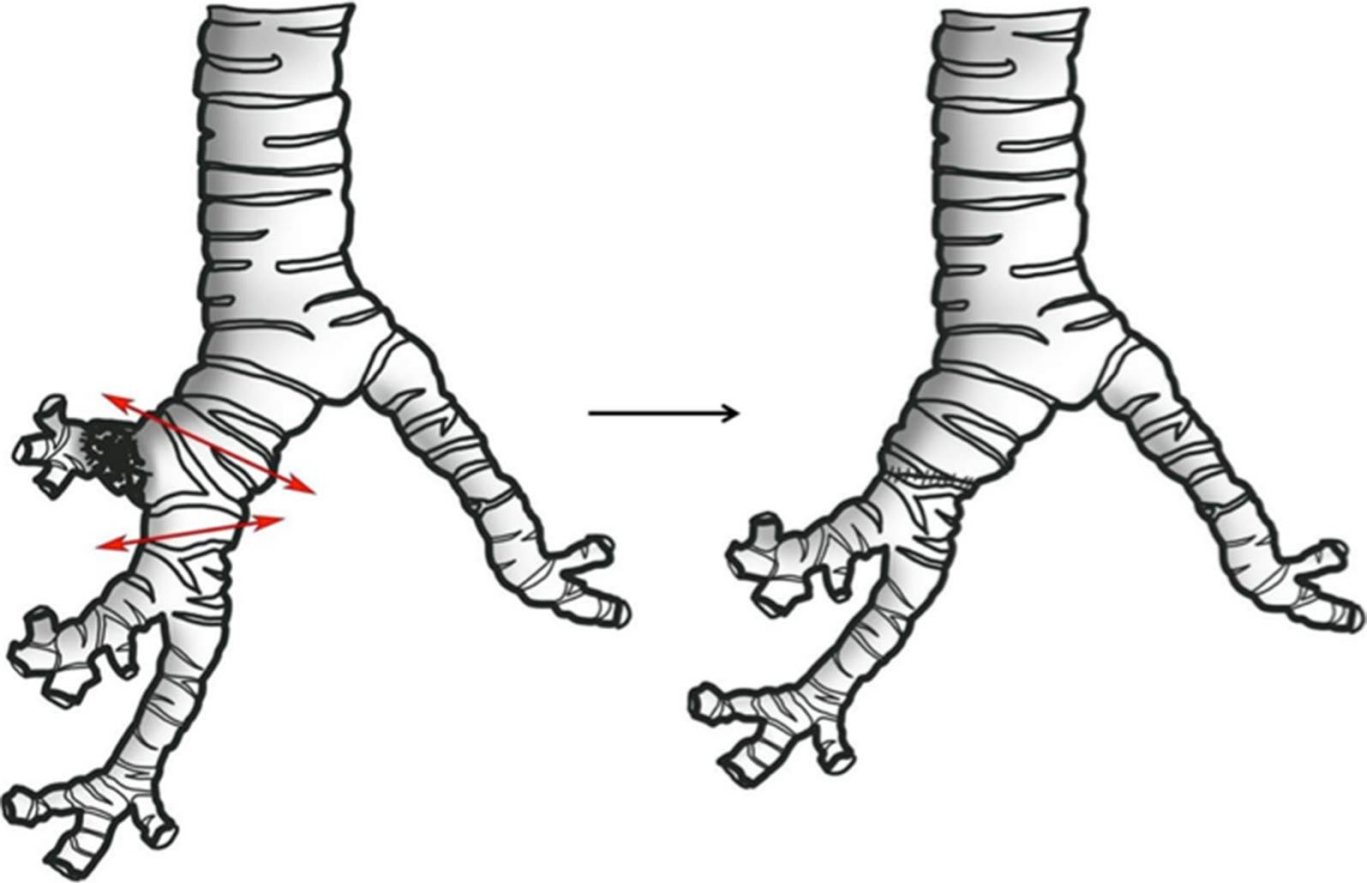
Schematic figure of sleeve lobectomy. Left: tumour involving the proximal right upper lobe bronchus is resected along with a segment of the intermediate bronchus with negative margins along with a segment of the intermediate bronchus. Right: termino-terminal anastomosis is achieved

### Complications

**Table 1:**
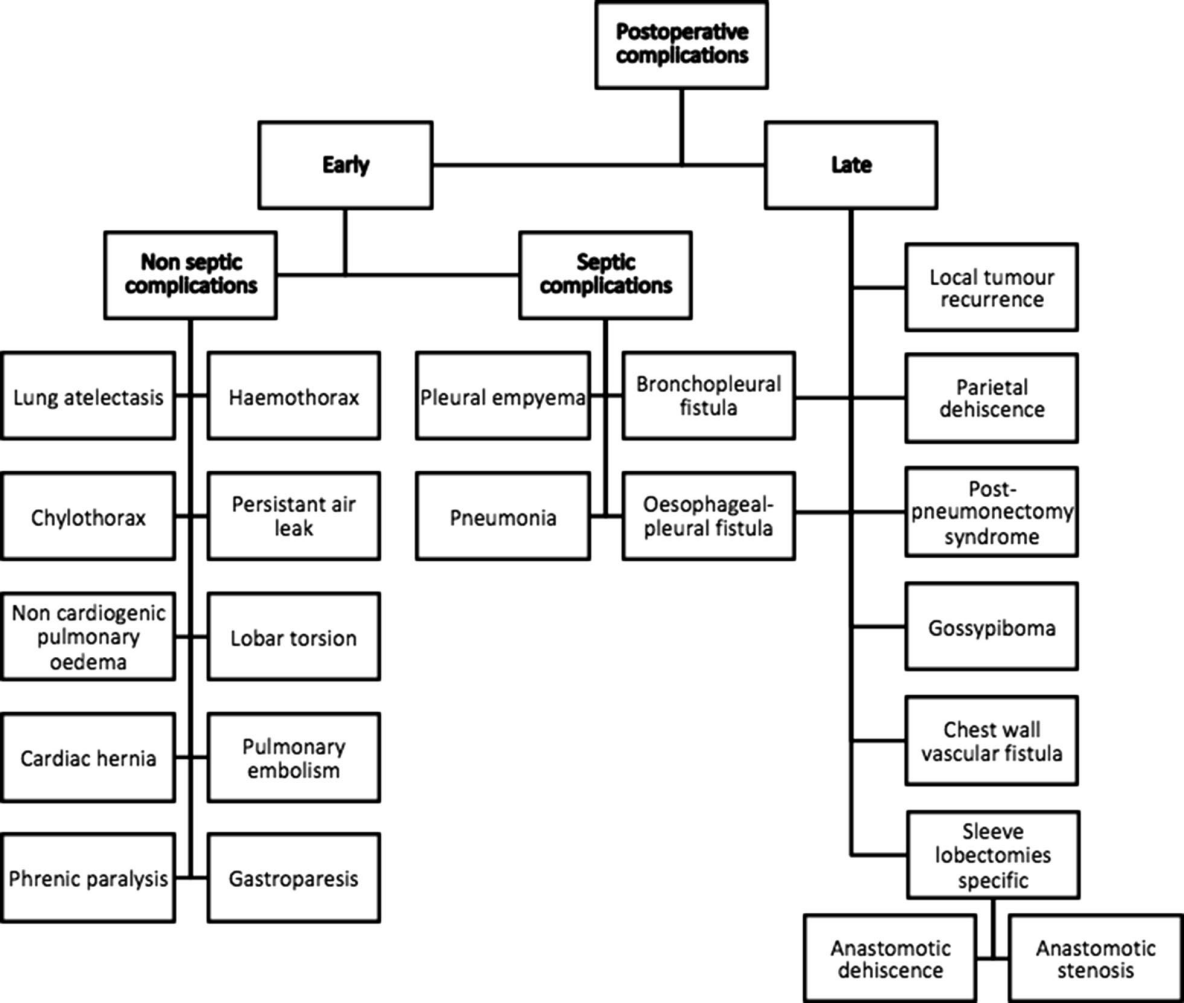
Summary of post-operative complications after thoracic surgery for lung cancer

### Early complications

**Fig. 8:**
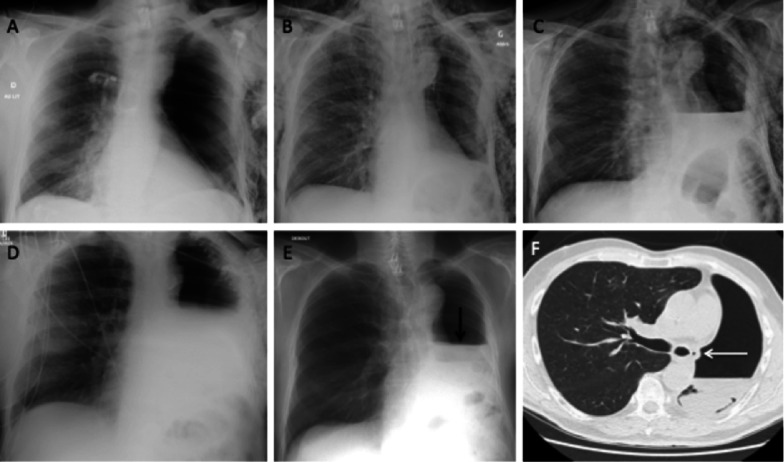
Bronchopleural fistula. **A**–**D** CXR 1 day, 3 days, 7 days and 10 days after surgery, with progressive elevation of the air–fluid level. **E** Febrile acute respiratory distress at day 28, CXR showing a decrease in the air–fluid level (black arrow). **F** Axial CT image (parenchymal window) at day 28 confirming that the air–fluid level is too low at this stage and showing an air bubble in contact with the bronchial stump (white arrow)
  Two major complications can occur consisting of pleural empyema and flooding of the contralateral lung with residual fluid from the lobectomy pouch leading to alveolar filling and ARDS.
B. **Pleural empyema (1–10%)**
  This severe complication happens especially after pneumonectomy in the early post-operative period, later occurrence would raise concern about bronchopleural or oesophageal–pleural fistula. CXR may show a rapidly forming pleural collection with one or more air–fluid levels. The CT scan may show an inversion of the internal concavity of the pleural pouch which takes on the appearance of a biconvex collection, a thickening of the pleural pouch wall with parietal enhancement after contrast injection and air–fluid levels (Fig. 9) [18].

A. **Bronchopleural fistula (2–13%)**
  It occurs mostly after pneumonectomy, especially on the right side, probably due to the anatomical characteristics of the right main bronchus (larger size, lesser mediastinal coverage than the left main bronchus) [18]. It can occur immediately after surgery due to a suture release or later due to a tumour recurrence or empyema.
  The radiological presentation can take several forms: decrease, reappearance or non-appearance of the air–fluid level of the pneumonectomy cavity, persistence or aggravation of a pneumothorax, pneumomediastinum or subcutaneous emphysema; visible fistula (in about 50% of cases) (Fig. 8) [19].

**Fig. 9:**
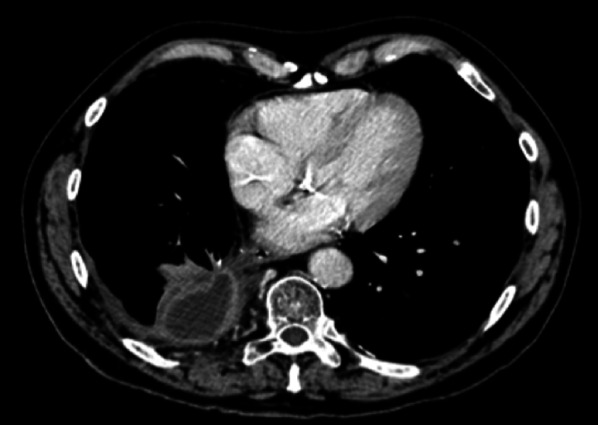
Pleural empyema. Axial post-contrast CT image (mediastinal window) showing a pyothorax caused by a bronchopleural fistula in the aftermath of a right upper lobectomy. We can observe loss of the internal concavity of the pleural pouch, with a biconvex collection, a thickening of the pleural pouch wall with parietal enhancement
C. **Lung atelectasis**
  Collapse is more frequent in early post-operative care and should raise the following hypotheses: mucous plug, blood clot, inflammatory or post-operative bronchial stenosis or bronchial torsion.
D. **Pneumonia (2–20%)**
  This diagnosis is often difficult and should not be based on CXR alone as a third of the located infiltrates on a CXR does not correspond to an infectious cause, and an unchanged CXR cannot exclude it. Confirmation can only be obtained by isolation of the germ after bronchoalveolar lavage. Two radiological patterns are possible: bronchopneumonia (blurred opacities of bilateral distribution, often associated with foci of centrilobular micronodules with tree-in-bud aspect) and lobar or segmental pneumonia, which are rarer. As imaging findings may not be specific, alternate diagnostics should be considered including amiodarone pneumopathy, often introduced for post-operative atrial fibrillation [20].
E. **Haemothorax (1.3%)**
  Haemothorax is rare, and many causes are possible: residual bleeding of systemic thoracic vessels (bronchial and intercostal arteries in particular), suture loosening of a pulmonary artery, venous wound [21]. Diagnosis is easier if the drains are still in place. After removal of the drains, a rapid increase in pleural effusion can be seen on CXR, in a context of acute anaemia; unenhanced CT may show a high attenuation pleural effusion (around 50 HU), heterogeneous or with a fluid–fluid level (Fig. 10). It is essential to look for extravasation of contrast agent or pulmonary arterial pseudoaneurysm for which embolisation is essential because of the risk of secondary rupture with haemorrhagic shock.

**Fig. 10:**
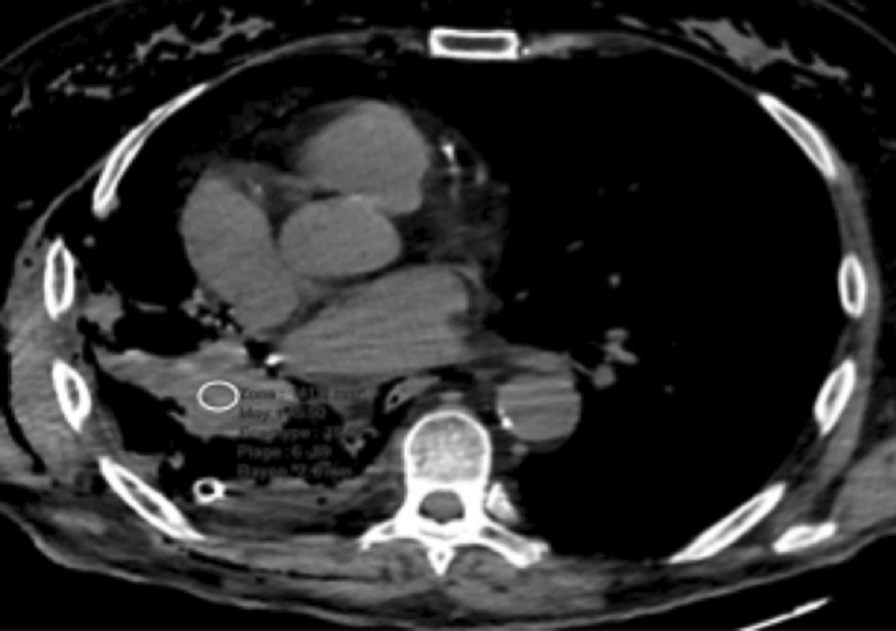
Haemothorax. Axial CT image (mediastinal window) showing haemothorax after right pneumonectomy. A region of interest placed in the effusion indicates a spontaneous density around 50 HU
F. **Chylothorax (1%)**
  A chylothorax can be the consequence of a wound to the thoracic duct or one of its main branches [22]. The radiological picture shows a too rapid filling of the pleural lodge, and an effusion whose attenuation is dependent on the protein concentration. If the concentration is high, the fluid is denser than water; rarely, a supernatant of fat density is present and can sustain the diagnosis. Pleural puncture, or aspiration for surgical drain bringing back a milky fluid, with measurement of triglycerides in the effusion (>110 mg/dl) confirms the diagnosis [23].
G. **Persistent air leak (3–8%)**
  Air leaks are considered persistent when they exceed 7 days after surgery, CXR typically shows the persistence of a pneumothorax, pneumomediastinum or subcutaneous emphysema. They are associated with increased morbidity rates and prolonged hospitalizations and are more frequent after wedge resections, lobectomies with incomplete fissure and in case of important emphysematous lesions [18, 24].
H. **Non-cardiogenic pulmonary oedema (2–15%)**
  It can occur after any type of resection but more frequently after right pneumonectomy, within 2–3 days after surgery. Vital prognosis is involved [22, 25]. The CT scan shows extensive ground-glass opacities with septal lines evolving towards gravito-dependent pulmonary condensations suggesting ARDS. Differential diagnoses include pulmonary oedema due to heart failure (importance of biology, associated right heart failure signs and echocardiography), lung infections (importance of the evolution and possible isolation of the germ by bronchial-alveolar lavage), re-expansion pulmonary oedema and aspiration pneumopathy (fistula) (Fig. 11).

**Fig. 11:**
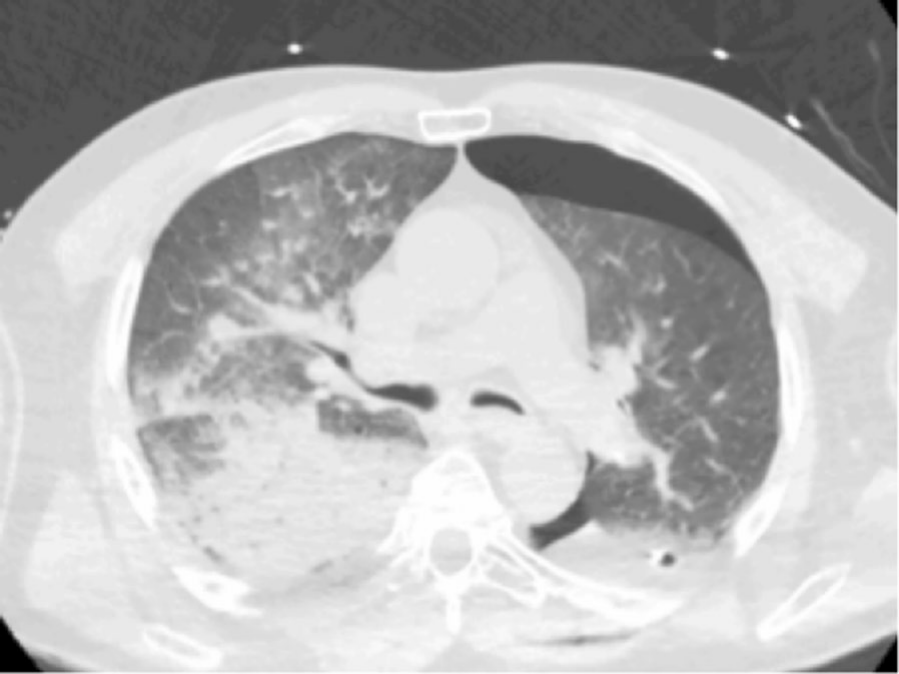
Non-cardiogenic pulmonary oedema. Axial CT image (parenchymal window) showing an ARDS after left lower lobe segmentectomy. In this case, ARDS abnormalities were predominant on the right side; gravito-dependent pulmonary condensations are present with normal anterior lung, then ground-glass opacities, then posterior alveolar condensations
I. **Lobar torsion (< 0.4%)**
  Also called twist, it is the torsion of a lobe adjacent to a lobectomy. In 70% of cases, this complication involves the middle lobe after right upper lobectomy. The torsion of the arterial, venous and bronchial pedicle may be more or less complete. Spontaneous evolution with haemorrhagic infarction and gangrene can be lethal [18, 26]. CXR shows condensation and abnormal displacement of the affected lobe. CT finds condensation of the lobe, with slight enhancement after injection, and torsion of the pedicle (Fig. 12).

**Fig. 12:**
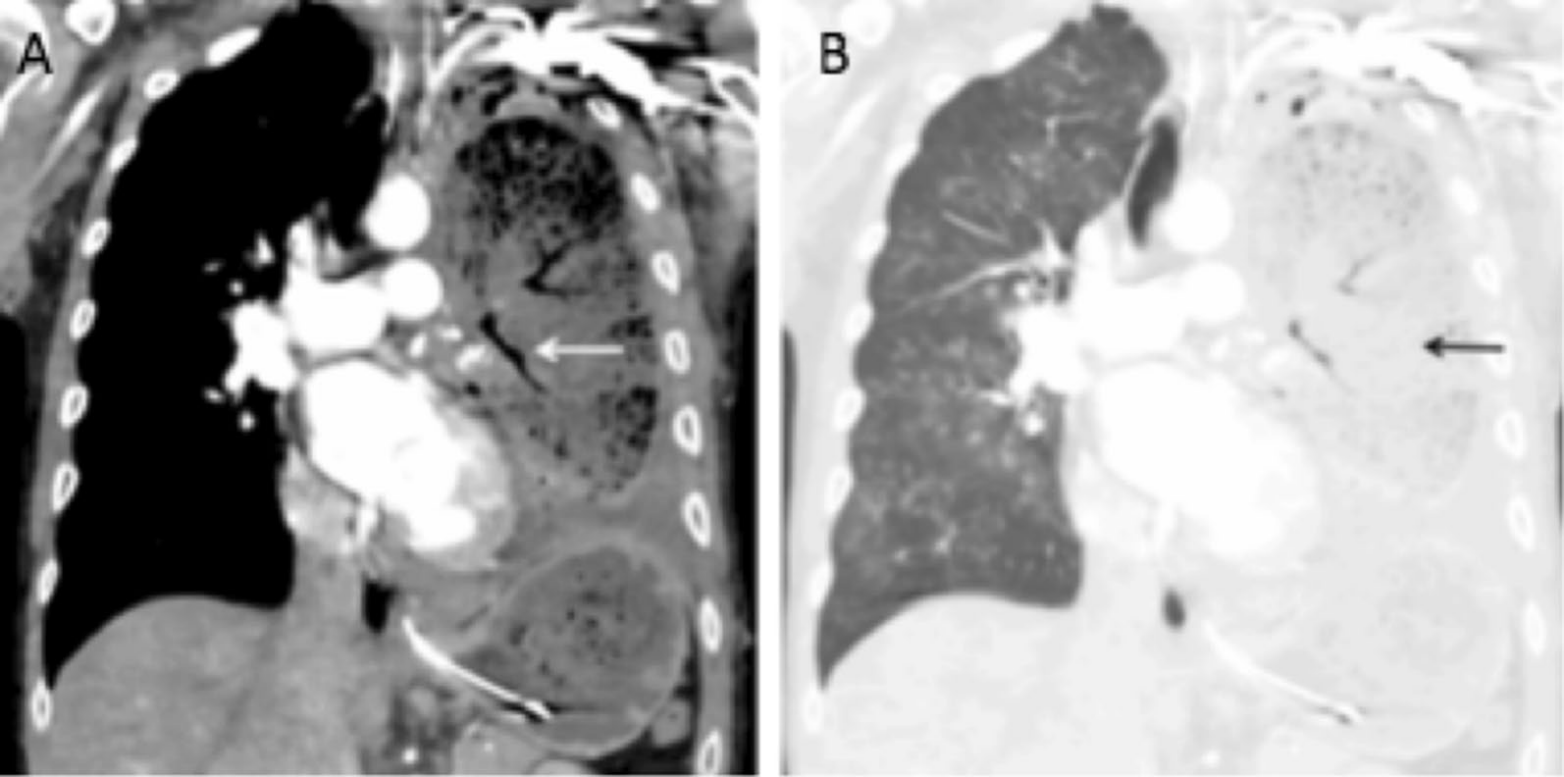
Lobar torsion. Coronal CT images (**A**: mediastinal window, **B** parenchymal window) showing a left lower lobar torsion after left upper lobectomy. **A** CT shows the torsion of the pedicle (white arrow) and the ascent of the left lower lobe. **B** We notice the condensation of the remaining lobe (black arrow)
J. **Cardiac hernia (< 0.1%)**
  This is the dislocation of the heart through a surgical pericardial breach, which occurs in the early post-operative period, resulting in cardiac decompensation. Chest X-ray and CT show a displacement of the heart in the pneumonectomy cavity. Treatment includes lateral decubitus on the side opposite the pneumonectomy and reoperation in extreme urgency [26].
K. **Oesophageal–pleural fistula (0.2–1%)**
  This complication is much rarer than bronchopleural fistula and can occur in the early post-operative period or later, leading to suspicion of recurrence [27, 28]. CT shows pleural empyema, and the fistula can be detected by ingestion of iodinated contrast medium (Fig. 13).

**Fig. 13:**
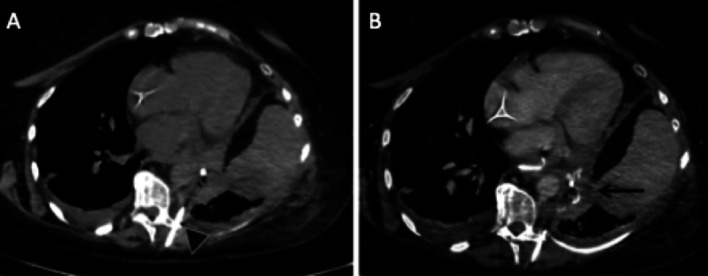
Oesophagopleural fistula. Axial CT images (mediastinal window) without injection and without ingestion of iodinated contrast medium (**A**), with injection and ingestion of iodinated contrast medium (**B**). We note the absence of spontaneous hyperdensity of the pleural empyema, and its filling with iodinated contrast medium after ingestion (black arrow). A drain was already in the empyema at the time of CT acquisition (black arrowhead)
L. **Pulmonary embolism**
  Although it is not different from conventional pulmonary embolism, it should not be confused with an alluvial thrombus within a pulmonary artery stump. This alluvial thrombus itself can be pathological when it is progressive (Fig. 14).

**Fig. 14:**
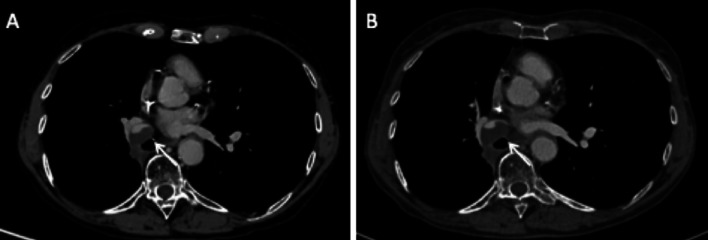
Progressive alluvial thrombus. Axial CT images (mediastinal window) in May 2019 (**A**) and November 2019 (**B**), showing an evolutive alluvial thrombus in the interlobar artery stump, respectively, 6 months and 11 months after right lower and middle bilobectomy
M. **Phrenic paralysis**
  Phrenic paralysis results from the intraoperative sectioning of the phrenic nerve, involuntary or more often necessary due to its infiltration by the tumour. It is suspected when the diaphragmatic dome rises. Confirmation can be obtained by « Sniff test » under fluoroscopic control, ultrasound exploration or dynamic MRI (Fig. 15).

**Fig. 15:**
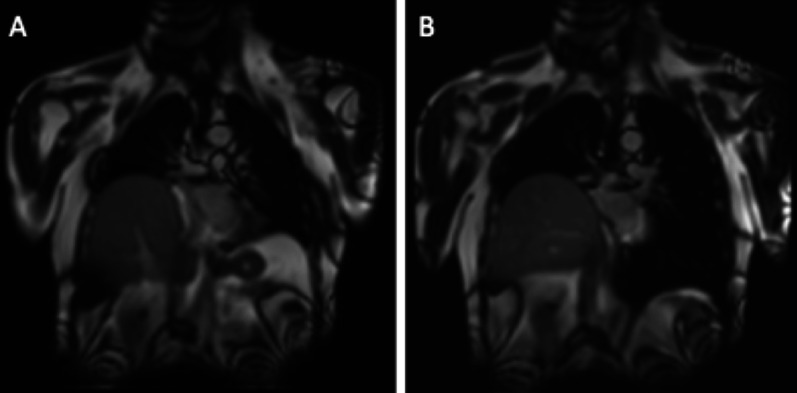
Phrenic paralysis. Coronal MR dynamic images showing a right phrenic paralysis after right lower lobectomy. During exhalation (**A**), the two diaphragmatic domes are raised; during inhalation (**B**), the left dome is lowered while the right remains in place
N. **Gastroparesis**
  It is a syndrome defined by an objectively delayed gastric emptying without mechanical obstruction. It results from a surgical disruption of the vagal pathways innervating the stomach and pylorus, involuntary or more often necessary. Gastroparesis should be suspected when early satiety, nausea, vomiting, abdominal pain and/or bloating occur, early after a surgery that may damage the vagus nerve. The diagnosis can be easily demonstrated on CT, by showing a marked gastric dilatation in the absence of mechanical obstruction or gastric masse (Fig. 16) [29].

**Fig. 16:**
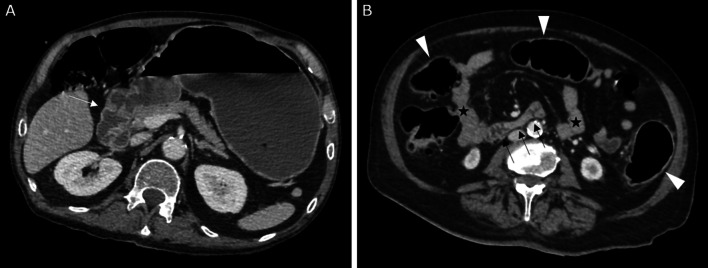
Gastroparesis. Axial CT images (portal venous phase) at the level of the pyloroduodenal junction (**A**) and of the third part of the duodenum (**B**), showing a marked gastric dilatation upstream of the pyloroduodenal junction (white arrow), without any mechanical obstruction. There is no duodenal dilatation (black arrows), neither of the other parts of the small intestine (black stars). Note the mild aeric colic distention (black arrowheads)

### Late complications

Note that bronchopleural fistula and oesophageal–pleural fistula can be both early and late complications.

**Fig. 17:**
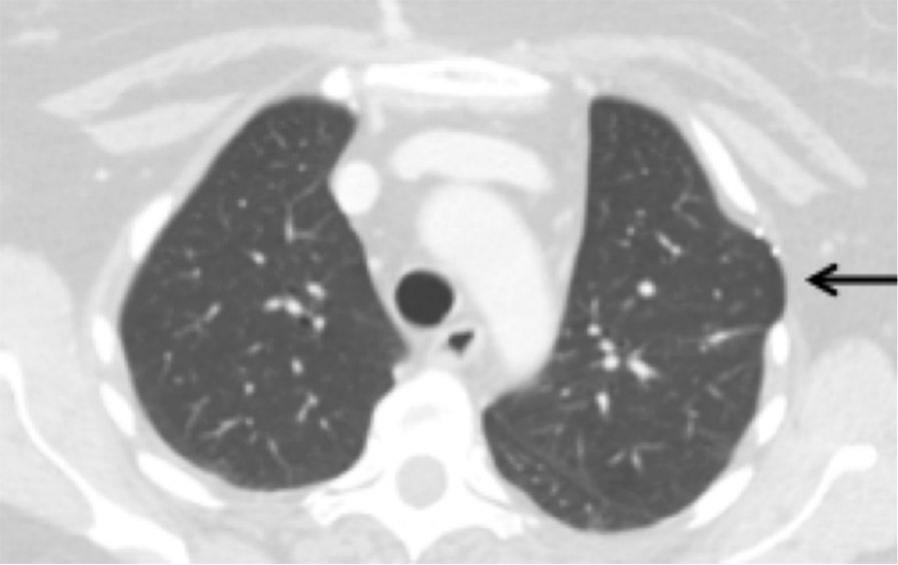
Parietal dehiscence. Left upper lobe parenchyma herniation (black arrow)
B. **Gossypiboma (< 0.1%)**
  Intrathoracic retention of an operating compress, which may be pleural, mediastinal or more rarely intrapulmonary after migration. It can result in a heterogeneous mass with air bubbles and sometimes calcifications, and a peripheral enhancement after injection. The use of radiolabelled compresses makes it possible to diagnose it on CXR [16].
C. **Post-pneumonectomy syndrome (0.1%)**
  This complication generally occurs lately, in patients operated on in childhood or adolescence, after complete resorption of the liquid contained in the pneumonectomy cavity, most often on the right side. This syndrome corresponds to the mediastinal displacement towards the side of the pneumonectomy, with stretching and compression of the bronchial tree against the descending aorta or the spine [19]. To diagnose dynamic compression, CT scan acquisitions can be done during exhalation as bronchial compression may sometimes be missed on inspiratory images alone. Moreover, evaluation of subsequent air trapping can be achieved on end-expiratory images.
D. **Chest wall vascular fistula (< 0.1%)**
  Very rare, this complication is the consequence of the development of systemic arterial fistulas in areas of pleural adhesions [19]. CT finds an irregular aspect of the pleura with an increase in the calibre of the systemic arteries (thoracic and intercostal); exceptionally, the fistula is visualised between the systemic arteries and the pulmonary veins or arteries flowing in the opposite direction. Arterio-embolization gives variable results.
E. **Local tumour recurrence**
  Recurrence usually occurs in the first 2 years after surgery and is often located along parenchymal staples (Fig. 15), on a bronchial suture, on the pleura (Fig. 15) or the chest wall. Recurrence may also be related to regional lymph nodes. This recurrence may manifest in various ways ranging from asymptomatic findings at systematic chest CT to respiratory failure due to tracheal obstruction, bronchopleural or oesophageal–pleural fistula. PET scan is sometimes useful to differentiate a recurrence from post-operative changes (Fig. 18).

A. **Parietal dehiscence**
  It corresponds to the herniation of the pulmonary parenchyma through a fragile area of the wall due to a failure of parietal reconstruction. It occurs frequently and is often considered as a normal post-operative finding. It can be pathological when significant and symptomatic. The diagnosis is usually clinical and can be easily demonstrated on CT (Fig. 17).

**Fig. 18:**
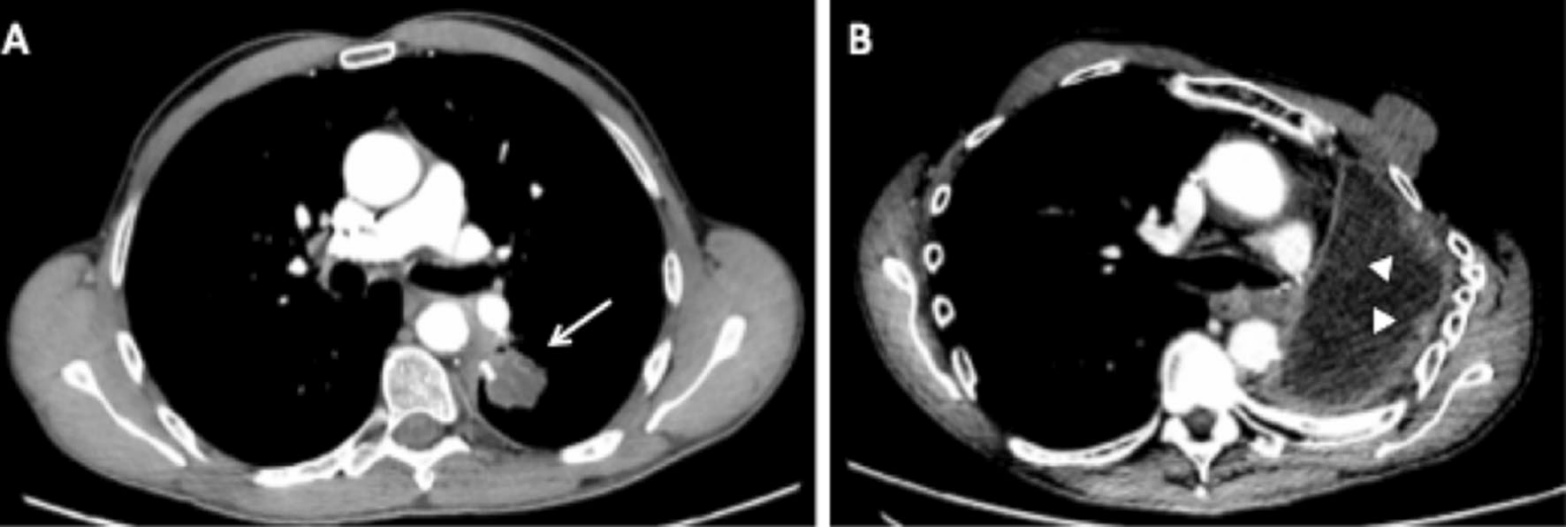
Local tumour recurrence. **A**: Axial CT image (mediastinal window) showing a tumour recurrence on a staple line from a left lower lobe wedge resection (white arrow). **B** Axial CT image (mediastinal window) showing a tumour recurrence on the pleura after left pneumonectomy (white arrowheads), 1 year after surgery
F. **Anastomotic dehiscence**
  This is a complication of sleeve lobectomies (6% of cases). It can be complicated by bronchopleural fistula and often requires a totalisation (pneumonectomy). CT can find a bronchial parietal defect at the site of the anastomosis (best sign but rarely found), pneumomediastinum, extra-mediastinal air bubbles surrounding the anastomosis, and bronchial narrowing and irregularity [30].
G. **Anastomotic stenosis**
  Late complication after sleeve lobectomy (18% of cases), it presents variable consequences depending on the degree of stenosis. It can be infra-clinical, responsible for air trapping or even collapse [30]. This stenosis can be visualised on CT.

## Conclusion

Surgical resection is the standard treatment for early and more advanced forms of non-small cell lung cancer. Surgical procedures include sublobar resection (wedge resection, segmentectomy), lobectomy, sleeve lobectomy and pneumonectomy. Each of these techniques can result in complex, but normal, post-operative findings. Knowledge of the normal post-operative anatomy, but also of the possible complications, helps the radiologist to participate in the reduction of post-operative morbidity and mortality, in both early and late post-operative periods.

## Data Availability

Not applicable. This is a review of publicly available information.

## Abbreviations

CT: Computed tomography
CXR: Chest radiograph
MIP: Maximum intensity projection
MinIP: Minimum intensity projection
